# Defining a highly conserved cryptic epitope for antibody recognition of SARS-CoV-2 variants

**DOI:** 10.1038/s41392-023-01534-0

**Published:** 2023-07-08

**Authors:** Aihua Hao, Wenping Song, Cheng Li, Xiang Zhang, Chao Tu, Xun Wang, Pengfei Wang, Yanling Wu, Tianlei Ying, Lei Sun

**Affiliations:** 1 MOE/NHC/CAMS Key Laboratory of Medical Molecular Virology, Shanghai Institute of Infectious Disease and Biosecurity, Shanghai Frontiers Science Center of Pathogenic Microorganisms and Infection, Shanghai Engineering Research Center for Synthetic Immunology, Shanghai Fifth People’s Hospital, Institutes of Biomedical Sciences, School of Basic Medical Sciences, Fudan University, Shanghai, China; 2 Biomissile Corporation, Shanghai, China; 3 State Key Laboratory of Genetic Engineering, Shanghai Institute of Infectious Disease and Biosecurity, School of Life Sciences, Fudan University, Shanghai, China

**Keywords:** Infection, Molecular medicine

**Dear Editor**,

In recent months, additional Omicron variants of severe acute respiratory syndrome coronavirus 2 (SARS-CoV-2), including the BA.5 sublineage BF.7 and BQ.1.1 and the BA.2 linage recombination XBB, XBB.1.5 and XBB.1.16, have emerged following the BA.2 and BA.5 subvariants. Remarkably, BQ.1.1 and XBB showed substantial neutralization escape as compared with the previous variants, and have gradually become the dominant variants worldwide.^1^ These facts emphasize the urgency of identifying highly conserved SARS-CoV-2 epitopes, which would be essential for the design of universal vaccines and broadly neutralizing antibodies.

We first explored whether a previously reported bispecific single-domain antibody bn03 could resist the immune escape of the SARS-CoV-2 Omicron variants by neutralization and binding activities.^2^ As shown in Fig. 1a, bn03 could broadly neutralize all the six Omicron variants, with IC_50_ values of 0.69, 0.36, 1.50, 0.10, 0.59 and 0.74 μg/ml for BA.2, BA.4, BA.5, BF.7, BQ.1.1 and XBB, respectively, which is comparable to that of the five SARS-CoV-2 variants of concern (VOCs Alpha, Beta, Gamma, Delta and Omicron BA.1, ranging from 0.11–0.76 μg/mL^2^). Correspondingly, bn03 exhibited potent binding to the RBD of BQ.1.1 and XBB spikes (S) (Fig. 1b, c).

**Fig. 1:**
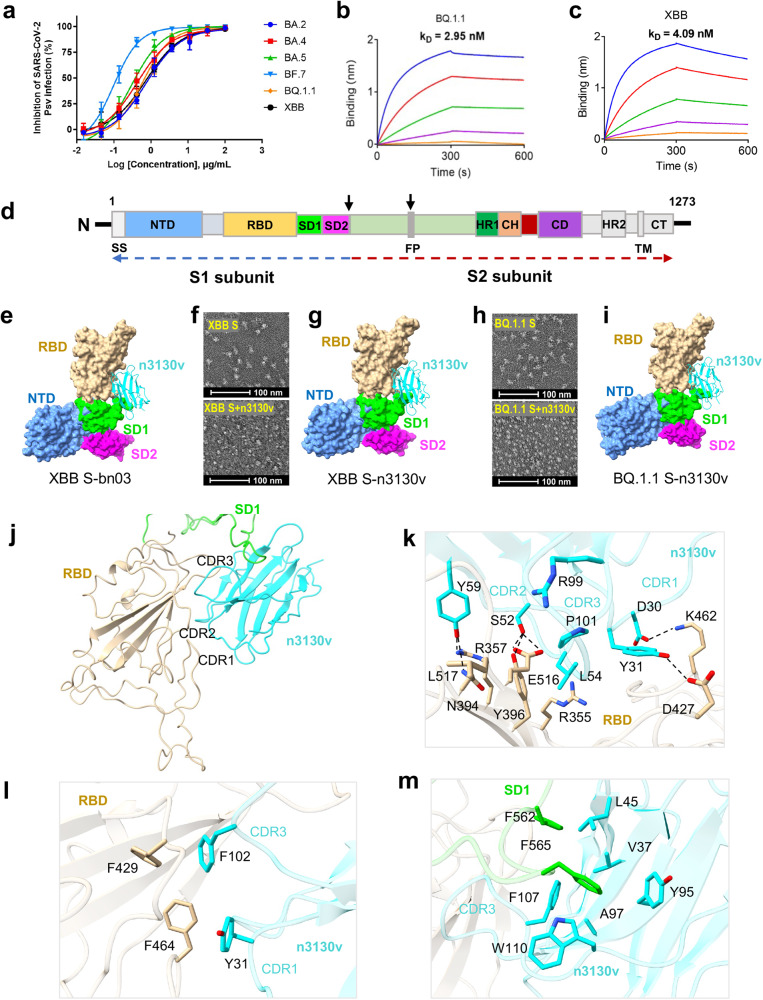
Cryo-EM structures of Omicron variants S monomer with bn03 and n3130v. **a** Neutralizing potency of bn03 against pseudoviruses of six Omicron subvariants. Three independent experiments were performed in triplicate. Binding affinity of bn03 to RBD of BQ.1.1 (**b**) and XBB (**c**) spikes. The K_D_ values are shown. **d** Schematic of SARS-CoV-2 Omicron S primary structure colored by domain. **e** The cryo-EM structure of XBB S-bn03 monomer, with XBB-S and n3130v displayed in molecular surface and cartoon modes. The n3130v and NTD, RBD, SD1, SD2 of XBB S are displayed in cyan, cornflower blue, tan, lime and magenta, respectively. **f** Negative staining EM images of XBB S-n3130v complex, showing that incubating n3130v with XBB S induced S trimer disassembling. **g** The cryo-EM structure of XBB S-n3130v. **h** Negative staining images of BQ.1.1 S-n3130v complex. **i** The cryo-EM structure of XBB S-n3130v. **j** The interactions between XBB S and n3130v. **k** The detailed interactions between XBB RBD and n3130v. The residues participating in the interactions are represented as sticks. A salt bridge and hydrogen bonds are indicated as dotted lines. **l** Hydrophobic interface between XBB RBD and n3130v. **m** Hydrophobic groove between XBB SD1 and n3130v

Analysis of mutational events across SARS-CoV-2 variants revealed that the n3130v domain of bn03 contributed to its exquisite neutralizing breadth (Supplementary Fig. 1). This domain was found to recognize a cryptic region located in the spike trimeric interface.^3^ Therefore, to precisely decipher the conserved epitopes, we not only determined the cryoelectron microscopy (cryo-EM) structure of bn03 in complex with the prefusion-stabilized SARS-CoV-2 Omicron XBB spike, but also the complexes of n3130v with spikes of XBB and BQ.1.1. In contrast to the apo-state spike trimers which mostly have one-RBD up and two-RBD down (UDD), two states of XBB spike were observed when incubated with bn03 for ten minutes: the spike trimers with one RBD up (UDD) and mostly, the spike monomer in complex with bn03 (XBB S-bn03) (Supplementary Figs. 2b, 3). Therefore, bn03 binding tends to disassemble the trimers into monomers. After an hour of incubation with bn03, most S trimers were disassembled (Supplementary Fig. 2b). Similarly, the mixture of n3130v with XBB (XBB S-n3130v) or BQ.1.1 spikes for an hour resulted in extensive trimer disassembly (Fig. 1f, h, Supplementary Fig. 2c, e). Such phenomenon was also observed in two other reported antibodies, S2H97 and 553–49, that target epitopes buried inside the spike trimer.^4,5^ Taken together, these results clearly demonstrate spike trimer disassembly as mechanism of action for the antibodies targeting trimeric interface epitopes.

In XBB S-bn03 complex, due to the flexibility of S monomer, the S1 (NTD-RBD-SD1-SD2) region and the n3130v domain of bn03 was locally refined to 3.98 Å resolution. The complex structures of XBB spike monomer-n3130v (XBB S-n3130v) and BQ.1.1 spike monomer-n3130v (BQ.1.1 S-n3130v) were determined to 3.44 Å and 3.68 Å, respectively (Supplementary Figs. 4, 5; Supplementary Table 1). The three complex structures revealed nearly identical binding epitopes that buried deep inside the trimeric interface of the spike proteins (Fig. 1d, e, g, i). A total of 38 residues from XBB S-RBD are involved, rendering 1391.6 Å^2^ buried surface area of the XBB spike monomer. These extensive contacts are mediated by both hydrophilic and hydrophobic interactions and all three CDRs of n3130v are involved (Fig. 1e, j). D30 of CDR1 forms a salt bridge with K462 of RBD. S52, L54 and G55 of CDR2 contacts R355, Y396, and E516 of RBD through six pairs of hydrogen bonds. R99 and P101 of CDR3 form three hydrogen bonds with L517 of RBD (Fig. 1k). F429 and F464 of RBD form a hydrophobic interface with Y31 and P102 of n3130v (Fig. 1i). Other than that, F562 and F565 of SD1 form another hydrophobic interface with V37, L45, Y95, A97, F107, W110 of n3130v (Fig. 1m), which is not observed in BA.1 S trimer-bn03 complex structure^4^ (PDB ID: 7WHK). Compared with BA.1 S trimer-bn03 complex, the S1 region of XBB S in S monomer-n3130v complex undergoes large conformational changes (Supplementary Fig. 6, Supplementary Video 1), inserting F562 and F565 residues of the SD1 domain into the hydrophobic groove of n3130v (Fig. 1m) and thus providing stronger interaction between S1 and 3130v. The n3130v epitopes on RBD and SD1 are highly conserved between the variants of SARS-CoV-2, including WT, BA.1, BQ.1.1, XBB, XBB.1.5 and the newly discovered XBB.1.16 (Supplementary Fig. 1). The highly conserved epitopes explain the exquisite neutralizing breadth of bn03. The relatively low immune pressure may contribute to the high conservation of such trimeric interface epitope.

In summary, our study defined a highly conserved epitope on the trimeric interface of viral spikes that enables broad antibody recognition of SARS-CoV-2 variants including the Omicron subvariants BQ.1.1 and XBB, and emphasized a unique mechanism for antibody neutralization via inducing SARS-CoV-2 spike trimer decay and disassembly.

## Supplementary information

Supplementary Material

Supplementary Video 1

## Data Availability

The cryo-EM maps and the coordinates of SARS-CoV-2 Omicron S variants complexed with bn03 or n3130v have been deposited to the Electron Microscopy Data Bank (EMDB) and Protein Data Bank (PDB) with accession numbers EMD-35170 and PDB 8I4E (XBB S-bn03), EMD-35171 and PDB 8I4F (XBB S-n3130v), EMD-35172 and PDB 8I4G (BQ.1.1 S-n3130v), EMD-35173 and PDB 8I4H (BA.1 S-bn03).
