# Supplementary material for: Defining a highly conserved cryptic epitope for antibody recognition of SARS-CoV-2 variants
Source: Signal Transduct Target Ther. 2023 Jul 8;8:269. doi: 10.1038/s41392-023-01534-0 (PMC10329685; doi:10.1038/s41392-023-01534-0)
Supplement: Supplementary file 1 — Supplementary Material [file 41392_2023_1534_MOESM1_ESM.doc]

**Supplementary Information**

**Defining a highly conserved cryptic epitope for antibody recognition of SARS-CoV-2 variants**

Aihua Hao^1#^, Wenping Song^1#^, Cheng Li^1#^, Xiang Zhang^1^, Chao Tu^2^, Xun Wang^3^, Pengfei Wang^3^, Yanling Wu^1^*, Tianlei Ying^1^*, Lei Sun^1^*

^1^MOE/NHC/CAMS Key Laboratory of Medical Molecular Virology, Shanghai Institute of Infectious Disease and Biosecurity, Shanghai Frontiers Science Center of Pathogenic Microorganisms and Infection, Shanghai Engineering Research Center for Synthetic Immunology, Shanghai Fifth People's Hospital, Institutes of Biomedical Sciences, School of Basic Medical Sciences, Fudan University, Shanghai, China;

^2^Biomissile Corporation, Shanghai, China;

^3^State Key Laboratory of Genetic Engineering, Shanghai Institute of Infectious Disease and Biosecurity, School of Life Sciences, Fudan University, Shanghai, China.

^#^These authors contributed equally to this work.

*Correspondence: Yanling Wu ([yanlingwu@fudan.edu.cn](mailto:yanlingwu@fudan.edu.cn)), Tianlei Ying ([tlying@fudan.edu.cn](mailto:tlying@fudan.edu.cn)), or Lei Sun ([llsun@fudan.edu.cn](mailto:llsun@fudan.edu.cn))

**Supplementary information include:**

Materials and Methods

Supplementary Fig 1 to 6

Supplementary Table 1

Supplementary Video 1

**Materials and Methods**

**Protein expression and purification**

The Fc fragment of human IgG1 was fused to the single-domain antibody of n3130v and cloned into mammal expression vector pSecTag2 containing an N-terminal murine Igκ chain leader sequence. The plasmid was transfected into HEK293 cells and incubated for 4 days at 37°C. The supernatant with secreted protein was collected and purified by Protein G (GenScript) according to the manufacture’s protocol. Sodium dodecylsulfate polyacrylamide gel electrophoresis (SDS-PAGE) was used to analyze protein integrity. The antibody bn03 was produced and provided by Wuxi Biologics.

**Bio-layer interferometry (BLI) binding assay**

The binding of antibodies to XBB and BQ.1.1 RBD proteins (Acro Biosystems) was measured by Octet-RED96 (ForteBio). The Ni-NTA biosensors was loaded by the his-tagged RBD (10 mg/mL), and incubated with antibodies in PBST for 300 s for association. The antibodies were three-fold serially diluted starting at 333 nM. Then, the sensors were immersed into PBST for 300 s. The curves were ﬁtted using the Data Analysis software 10.0 by 1:1 binding model. K_D_ values were determined with R^2^ values of higher than 95% conﬁdence level.

**Validation of SARS-CoV-2 pseudovirus**

All the SARS-CoV-2 subvariants spike pseudovirus (include BA.2 (PSV017), BA.4 (PSV022), BA.5 (PSV023), BF.7 (PSV026), BQ.1.1 (PSV028) and XBB (PSV030) were purchased from Sino Biological Inc. Pseudoviruses were ten-fold serially diluted and used to infect 293T/ACE2 cells. The infectivity of pseudovirus was determined by luciferase activity.

**Pseudovirus neutralization assay**

293T/ACE2 cells were coated in 96-well plate to form monolayer cells. Pseudovirus was mixed with antibody (three-fold serially diluted) at ratio of 1:1 at 37℃ for 1 h. The mixture was incubated with 293T/ACE2 cells. After 12 hours, the culture was refreshed with DMEM supplied with 10% FBS. After 48 hours, the cells were washed with PBS, and lysed with 1× lysis reagent (Promega). RLU readout was detected using Fireﬂy Luciferase Assay Kit (Promega). IC_50_ was calculated by the four parameters regression equation using GraphPad Prism.

**Expression and purification of SARS-CoV-2 XBB and BQ.1.1 Spike**

The gene encoding XBB and BQ.1.1 S ectodomain was purchased from GenScript. The XBB 6P substitution^1^ was inserted into vector pcDNA3.1. The plasmids were transfected into HEK293F cells using polyethlenimine. The supernatants were harvested after 72 h, and then purified by Histrap HP column (GE Healthcare). The protein was further purified on a Superose 6 increase 10/300 column (GE Healthcare) in 20 mM Tris, 200 mM NaCl, pH 8.0.

**Formation of XBB S-bn03, XBB S-n3130v and BQ.1.1 S-n3130v**

The bn03 was mixed with the purified XBB S trimer at 0.5 mg/mL in a 2:1 molar ratio, and incubated on ice for 10 min. The n3130v-Fc was mixed with XBB S trimer or BQ.1.1 S trimer in a 2:1 molar ratio, incubated at 4℃ for 1 h, and further purified on a Superose 6 increase 10/300 column (GE Healthcare) in 20 mM Tris, 200 mM NaCl, pH 8.0. The peak fraction was concentrated to 0.5 mg/ml.

**Cryo-EM sample preparation**

A 3 μL of XBB-S-bn03, XBB S-n3130v or BQ.1.1 S-n3130v complex sample was deposited on a glow-discharged holey amorphous nickel-titanium alloy film supported by 400-mesh gold grids. The sample was blotted away for 2 seconds with a force of -3 before being plunge frozen into liquid ethane using the Vitrobot IV (FEI/Thermo Fisher Scientific).

**Cryo-EM data collection and image processing**

For XBB S-bn03, XBB S-n3130v and BQ.1.1 S-n3130v, cryoEM data were collected on a Titan Krios G4 microscope operated at 300 kV, equipped with a Falcon 4i and a Seletrics X Imaging filter (Thermo Fisher) setting to a slit width of 20 eV. EPU software was used for automated data acquisition.

The video was taken in AFIS mode at a nominal magnification 130,000×, corresponding to a physical pixel size of 0.932 Å, and a defocus range from −1.0 μm to −3.0 μm. Each EER movie stack was dose-fractionated to 1080 frames with a total exposure dose of about 50^e−^/Å^2^. For BA.1 S-bn03, cryo-EM data were the same one that had been published to reconstruct spike trimers combined with bn03^2^.

All the data processing was performed with a routine procedure as described before^2^. The statistics of these cryoEM data were listed in Table S1. The reported resolutions are based on the Fourier shell correlation (FSC) 0.143 criterion in cryoSPARC software (Supplementary Fig. 5c)^3^. The sharpened maps were generated by DeepEMhancer^4^ for model building and analysis.

**Model building and refinement**

For model building, swiss-model^5^ was used to get these variants of omicron Spike monomers. Bn03 or n3130v model in 7WHI was used to fitted into the maps using UCSF Chimera^6^, and manually adjusted with COOT^7^. Several iterative rounds of real-space refinement were further performed in PHENIX^8^. Model validation was carried out using MolProbity. Figures were prepared using UCSF Chimera and UCSF ChimeraX^9^.

**References:**

1 Hsieh, C. L. *et al.* Structure-based design of prefusion-stabilized SARS-CoV-2 spikes. *Science* **369**, 1501-1505 (2020).

2 Li, C. *et al.* Broad neutralization of SARS-CoV-2 variants by an inhalable bispecific single-domain antibody. *Cell* **185**, 1389-1401.e1318 (2022).

3 Punjani, A., Rubinstein, J. L., Fleet, D. J. & Brubaker, M. A. cryoSPARC: algorithms for rapid unsupervised cryo-EM structure determination. *Nat Methods* **14**, 290-296 (2017).

4 Sanchez-Garcia, R. *et al.* DeepEMhancer: a deep learning solution for cryo-EM volume post-processing. *Commun Biol* **4**, 874 (2021).

5 Waterhouse, A. *et al.* SWISS-MODEL: homology modelling of protein structures and complexes. *Nucleic Acids Res* **46**, W296-w303 (2018).

6 Pettersen, E. F. *et al.* UCSF Chimera--a visualization system for exploratory research and analysis. *J Comput Chem* **25**, 1605-1612 (2004).

7 Emsley, P., Lohkamp, B., Scott, W. G. & Cowtan, K. Features and development of Coot. *Acta Crystallogr D Biol Crystallogr* **66**, 486-501 (2010).

8 Afonine, P. V. *et al.* Real-space refinement in PHENIX for cryo-EM and crystallography. *Acta Crystallogr D Struct Biol* **74**, 531-544 (2018).

9 Pettersen, E. F. *et al.* UCSF ChimeraX: Structure visualization for researchers, educators, and developers. *Protein Sci* **30**, 70-82 (2021).


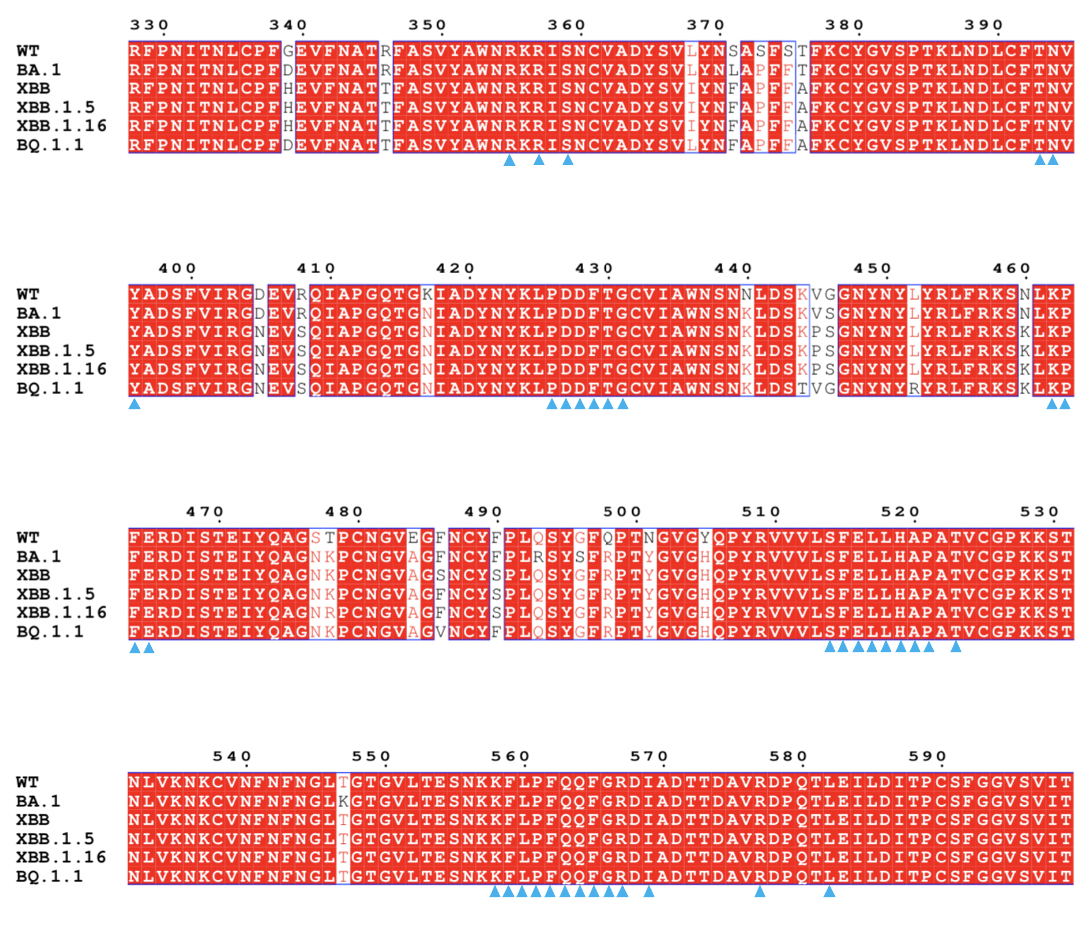


**Supplementary Fig 1. Sequence alignment of Omicron BA.1, XBB, XBB.1.5, XBB.1.16 and BQ.1.1.** Conserved amino acids are highlighted as red. Residues involved in n3130v binding are marked with blue triangles.

**
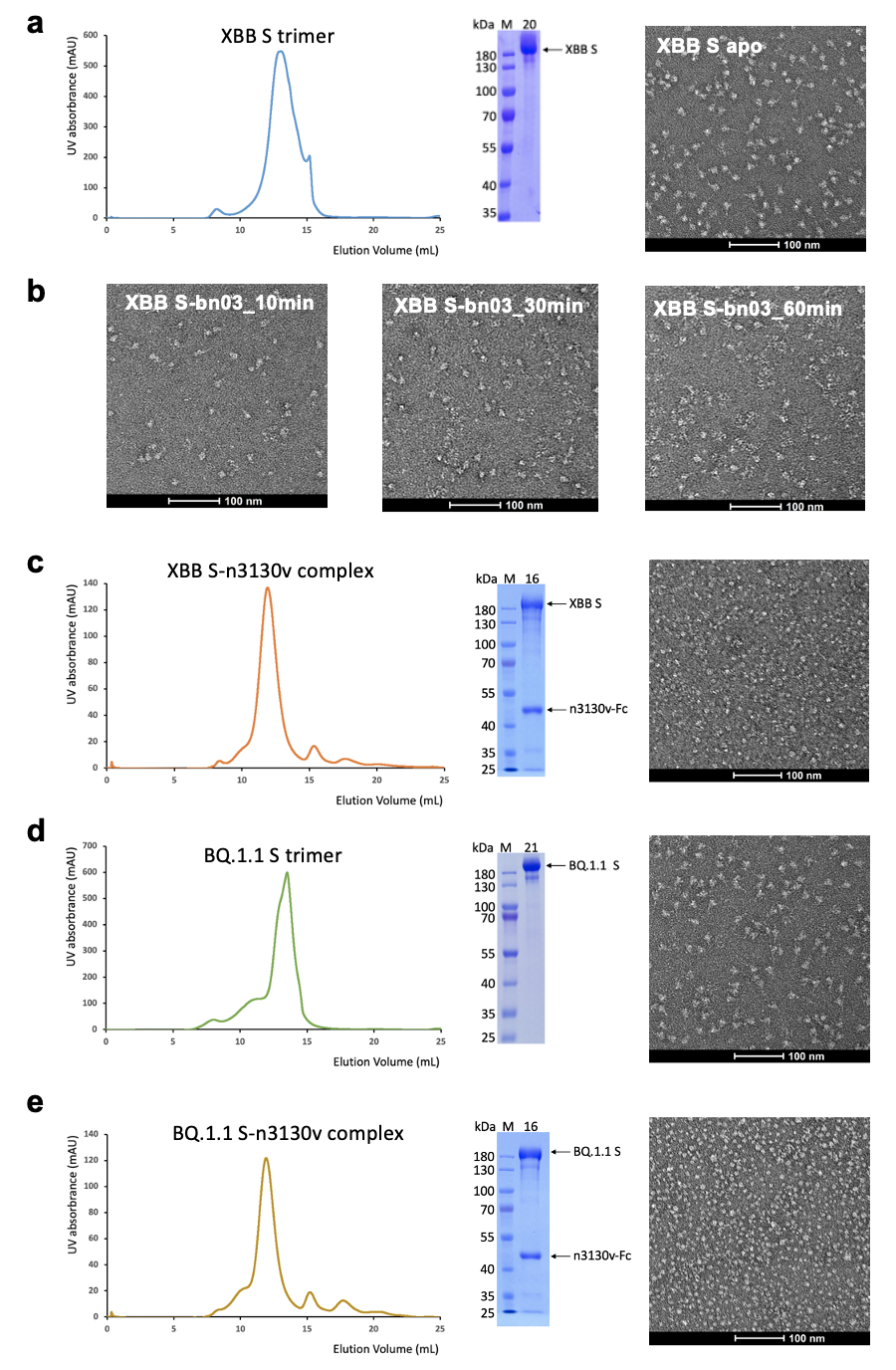
**

**Supplementary Fig 2. Sample purification. a** Purification and negative staining EM images of XBB S trimer. **b** Negative stain images of XBB S-bn03 complex, showing that incubating with bn03 leads to XBB S trimer disassembly. **c** Purification and negative staining EM images of XBB-n3130v complex. **d** Purification and negative staining EM images of BQ.1.1 S trimer. **e** Purification and negative staining EM images of BQ.1.1-n3130v complex.


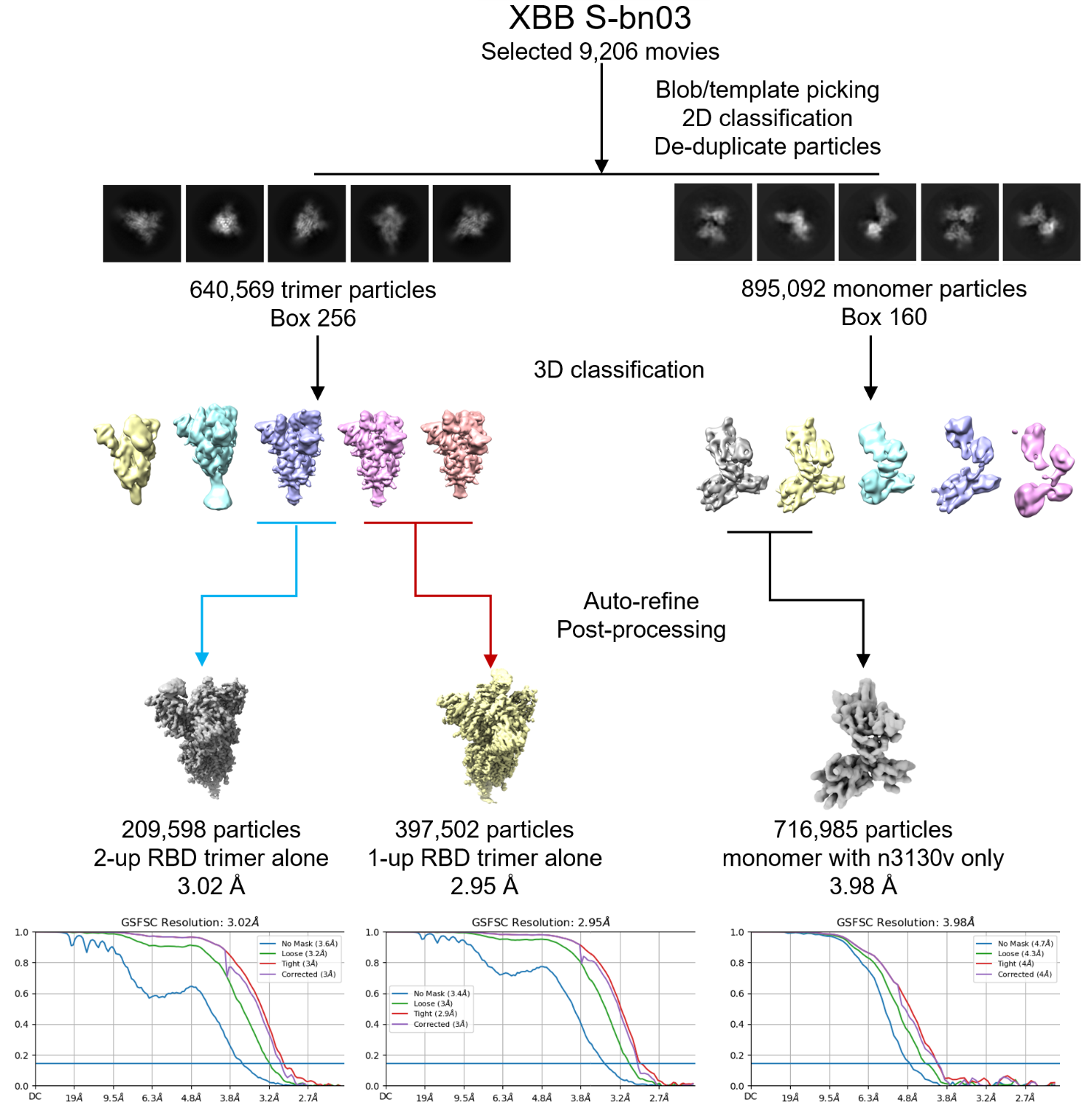


**Supplementary Fig 3. Cryo-EM data processing flowchart of XBB-bn03.**


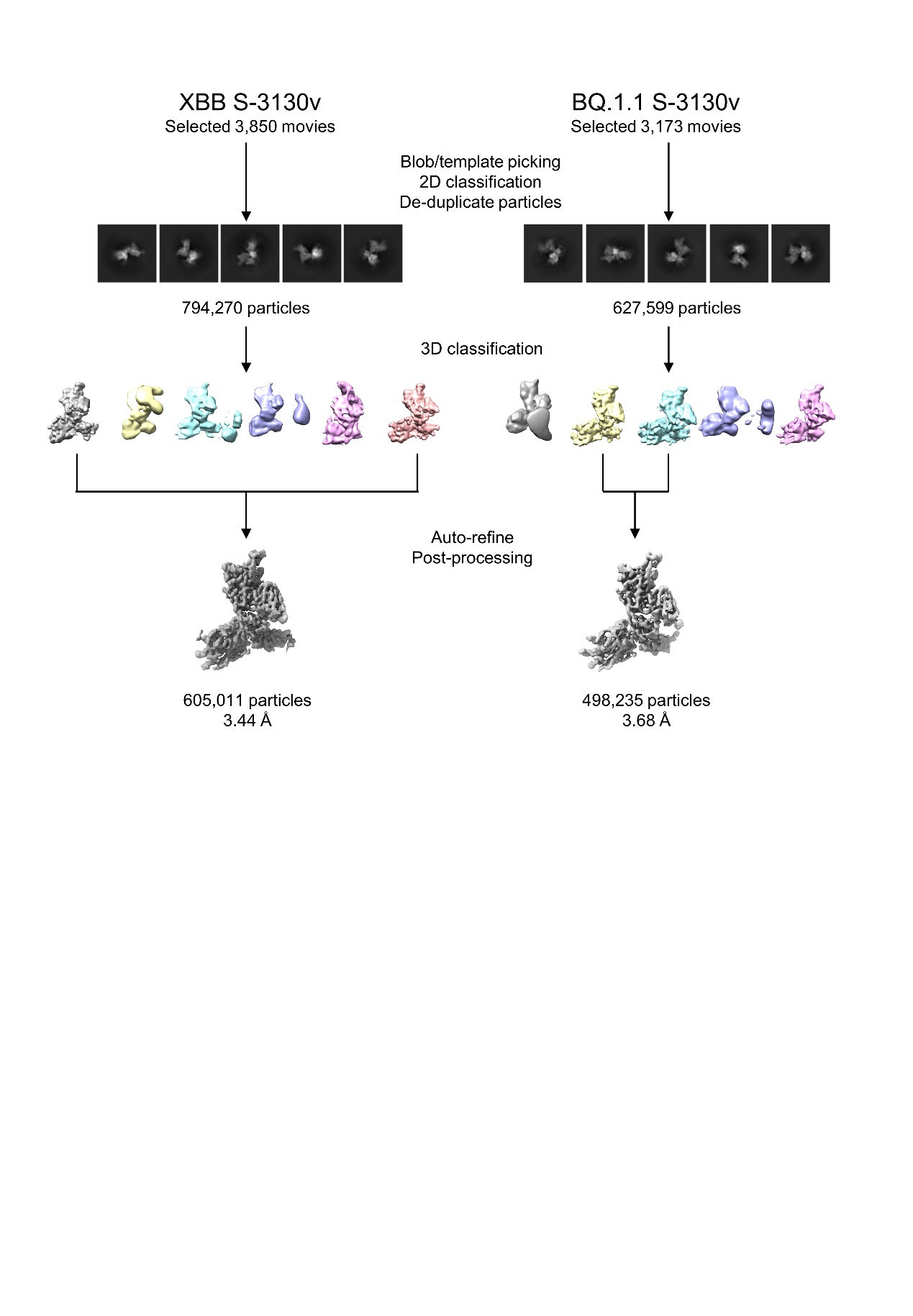


**Supplementary Fig 4. Cryo-EM data processing flowchart of XBB-n3130v and BQ.1.1-n3130v.**

**
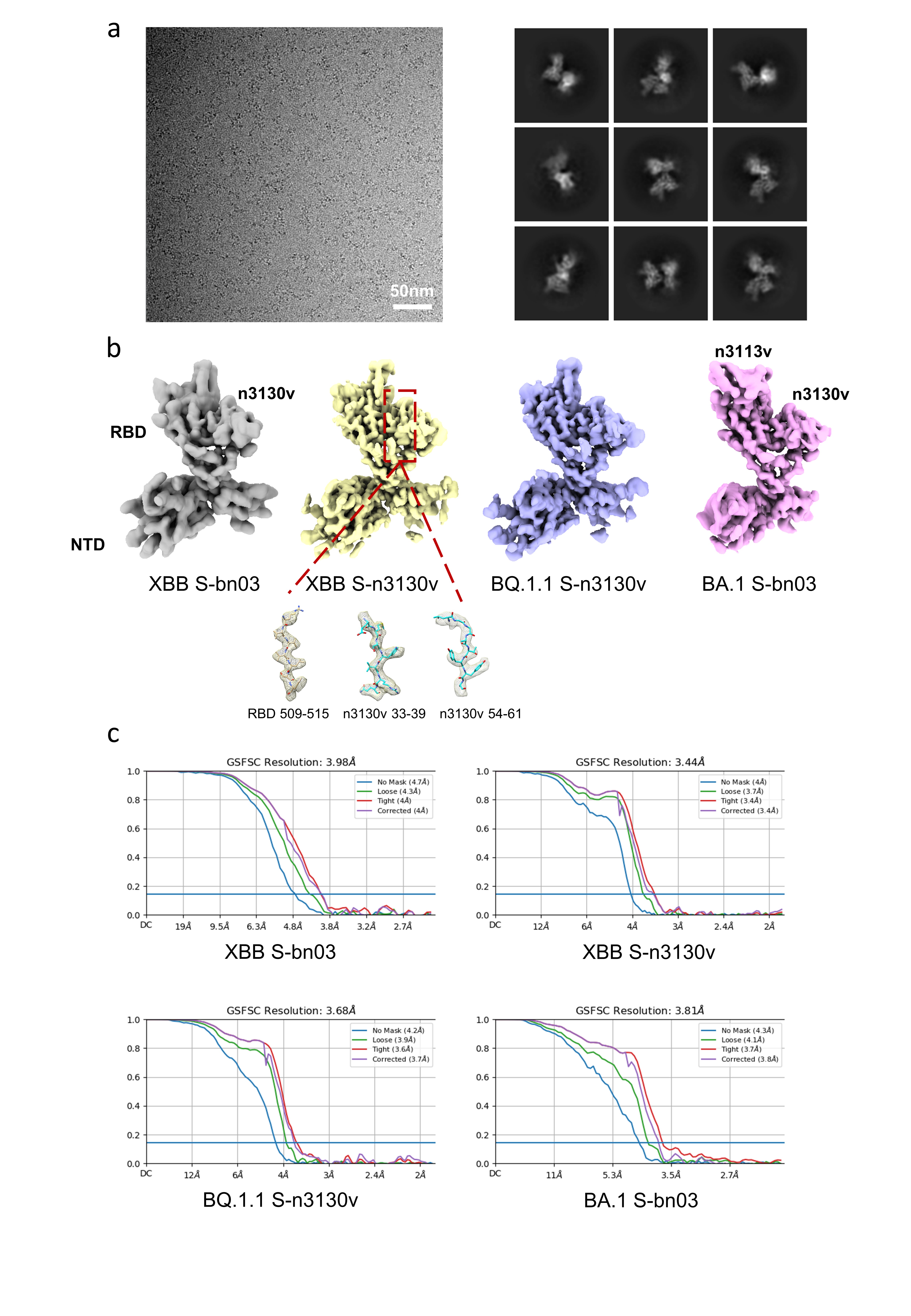
**

**Supplementary Fig 5. Cryo-EM results of Omicron spike variants with bn03 and n3130v-Fc. a** Representative electron micrograph and 2D classification results of XBB S-n3130v. **b** The reconstruction maps of the bn03 or n3130v-Fc with Omicron spike variants. **c** Gold-standard Fourier Shell Correlation (GSFSC) curves generated from cryoSPARC for each structure.


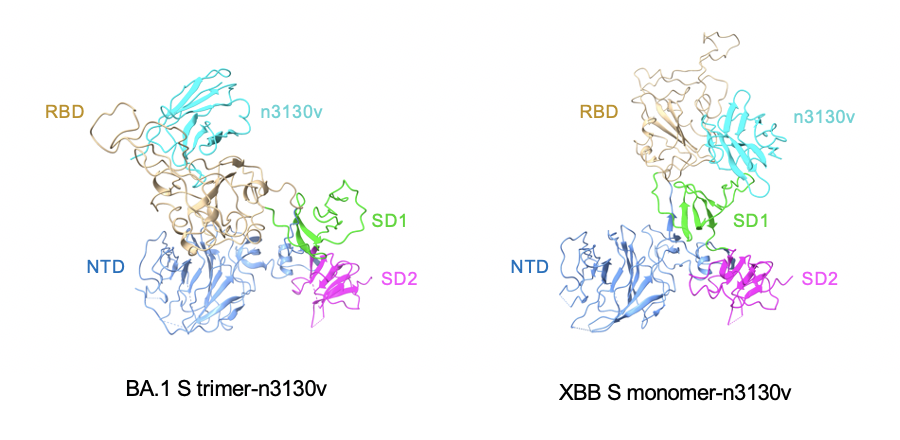


**Supplementary Fig 6. Conformational changes of the S1 region of XBB S monomer-n3130v complex compared with BA.1 S trimer-bn03 complex.** The NTD, RBD, SD1 and SD2 of BA.1 S or XBB S are displayed in ribbon mode colored in cornflower blue, tan, lime and magenta, respectively. N3130v is displayed in cyan ribbon.

**Supplementary Table 1 Cryo-EM data collection and refinement statistics**

| Protein sample | XBB S-bn03 | XBB S-n3130v | BQ.1.1 S-n3130v |
| --- | --- | --- | --- |
| PDBID  EMDB | 8I4E  EMD-35170 | 8I4F  EMD-35171 | 8I4G  EMD-35172 |
| Voltage (kV) | 300 | 300 | 300 |
| Detector | Falcon 4i | Falcon 4i | Falcon 4i |
| Movies | 9,206 | 3,850 | 3,173 |
| Pixel size (Å) | 0.932 | 0.932 | 0.932 |
| Electron dose (e^-^/Å^2^) | 50 | 50 | 50 |
| Defocus range (μm) | 1.0-3.0 | 1.0-3.0 | 1.0-3.0 |
| Symmetry | C1 | C1 | C1 |
| Final particles | 716,985 | 605,011 | 498,235 |
| Overall resolution (Å) | 3.98 | 3.44 | 3.68 |

**Model refinement and validation statistics**

| Ramachandran statistics | | |  |
| --- | --- | --- | --- |
| Favored (%) | 93.78 | 94.06 | 90.97 |
| Allowed (%) | 6.22 | 5.94 | 9.03 |
| Outliers (%) | 0.00 | 0.00 | 0.00 |
| MolProbity score | 2.53 | 2.41 | 2.48 |
| Rotamer outliers (%) | 6.84 | 5.29 | 5.71 |
| R.m.s.d | | |  |
| Bond lengths (Å) | 0.002 | 0.002 | 0.002 |
| Bond angles (^o^) | 0.424 | 0.442 | 0.622 |
|  |  |  |  |

**Supplementary Video 1. Conformational changes of the S1 region of XBB S monomer-n3130v complex compared with BA.1 S trimer-bn03 complex.**
